# Microbial communities in peatlands along a chronosequence on the Sanjiang Plain, China

**DOI:** 10.1038/s41598-017-10436-5

**Published:** 2017-08-29

**Authors:** Xue Zhou, Zhenqing Zhang, Lei Tian, Xiujun Li, Chunjie Tian

**Affiliations:** 1 Key Laboratory of Mollisols Agroecology, Northeast Institute of Geography and Agroecology, Chinese Academy of Sciences, Changchun, 130102 China; 2 Key Laboratory of Wetland Ecology and Environment, Northeast Institute of Geography and Agroecology, Chinese Academy of Sciences, Changchun, 130102 China; 3 University of the Chinese Academy of Sciences, Beijing, 100049 China

## Abstract

Microbial communities play crucial roles in the global carbon cycle, particularly in peatland ecosystems under climate change. The peatlands of the Sanjiang Plain could be highly vulnerable to global warming because they are mainly located at the southern limit of northern peatlands. In this study, the alpha diversity and composition of bacterial communities in three different minerotrophic fens along a chronosequence were investigated. We captured a rich microbial community that included many rare operational taxonomic units (OTUs) but was dominated by a few bacterial classes that have frequently been detected in other peatland ecosystems. Notably, a large diversity of methanotrophs affiliated with Alpha- and Gammaproteobacteria was also detected. Bacterial alpha diversity and composition varied as a function of peat depth and its associated physical-chemical properties, such as total carbon, total nitrogen, pH and bulk density. We also found that bacterial community turnover (beta diversity) to be significantly correlated with soil age, whereas bacterial alpha diversity was not.

## Introduction

Despite covering only 6–8% of the world’s terrestrial ecosystems, northern peatlands store approximately 30% of the global soil carbon (C)^1^. Thus, as one of the largest atmospheric carbon sinks and CH_4_ sources, northern peatlands have played an important role in global C cycles and climate change throughout the Holocene (from approximately 11600 years ago to the present)^2, 3^. In peatlands, the sequestration of C arises from an imbalance between inputs via primary productivity and losses through microbial decomposition, which releases C to the atmosphere and as dissolved organic carbon (DOC) exports^4, 5^. Both environmental and physico-chemical conditions impact C cycling through primary production and decomposition processes. Methane production is a consequence of the permanently waterlogged condition of the peat layers, where anaerobic decomposition prevails, and many studies have shown that microorganisms play a crucial role in C decomposition and methane production process^6–10^. Although microbial communities in the peatlands of Europe, America, Canada and the UK have been investigated^11–13^. However, there are relatively few studies on the peatlands in more temperate regions^14–16^. The Sanjiang Plain, located in the temperate climate region, is the largest area of freshwater marshlands in China^17^. As these peatlands are mainly located at the southern limit of northern peatlands, they may be more vulnerable to global warming than other areas^18^. Thus, it is imperative that we deepen our understanding of the microorganisms in this ecosystem.

The peatlands of the Sanjiang Plain have developed under certain topographic conditions during the Holocene or earlier^19^, and they vary in age from 500 to 10,000 years^20^. Correspondingly, these areas differ with regard to C accumulation rate. Accordingly, this chronosequence of peatlands offers an exceptional opportunity for studying links between the chronological characterization (soil age), soil properties and microbial community structure in peatland ecosystems. The influences of vegetative communities, water hydrology and soil properties in peatland microbial communities have been examined in many studies^21–24^, though the association between chronological characterization and the distribution of the microbial community structure has been neglected to date.

In the present study, high-throughput Illumina sequencing of 16S rRNA genes was applied to examine the bacterial communities of the Sanjiang Plain, the southern edge of northern peatlands. Peat cores were collected from three fens that began to develop in this area during different periods. The greater sequencing depth achieved by high-throughput sequencing allows for the capture of the less abundant and uncultured taxa and thus supplies a more thorough characterization of peatland bacterial diversity. Moreover, such chronological characterization facilitates the description of potential links between bacterial communities and soil age as well as the C accumulation rates.

## Results

### Physico-chemical and chronological characterization of peat cores

**Figure 1:**
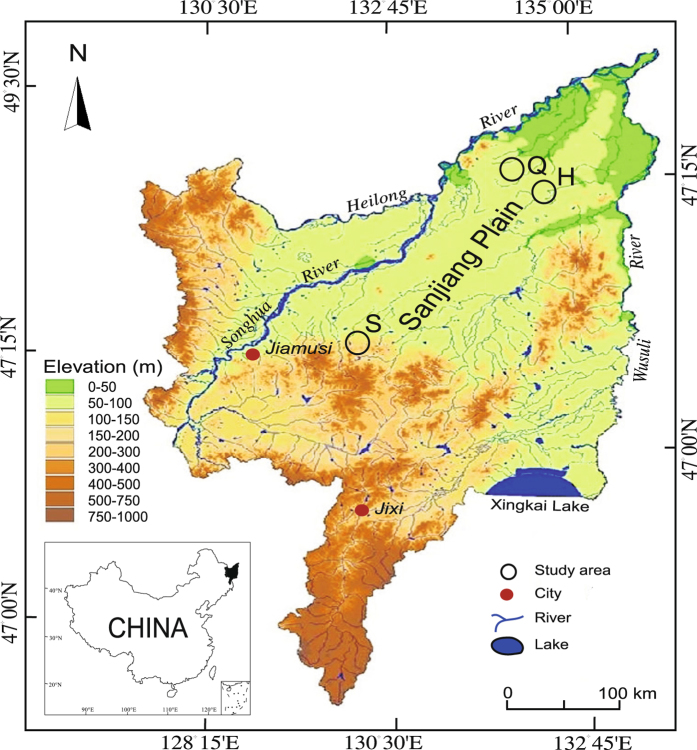
Map of the study region showing locations of the sampling sites on the Sanjiang Plain. The map (including the inset map of china) was generated by Zhenqing Zhang using ArcGIS 10.0 (http://www.esrichina.com.cn/softwareproduct/ArcGIS/).

Nine peat cores were retrieved from fens, S (Shenjiadian), H (Honghe), and Q (Qindelie), on the Sanjiang Plain, northeastern China (Fig. 1). With a depth of 100 cm, the peatlands located in Q are shallower than those at the other sites; mud deposits occur at depths greater than 100 cm. Accelerate mass spectrometry (AMS) dating results indicate that Sanjiang Plain peatlands began to develop over a wide range of dates during the Holocene (Fig. S1). The peat was dated from 637 to 11,496 cal. yr BP (Table 1). Peat cores from Q exhibited the most ancient chronological dates, with even the 30–60-cm section being dated to 3136 ± 1325 cal. yr BP, which was older than the age of the bottom section (100–200 cm) of the youngest fen, S at 2881 ± 1338.95 cal. yr BP, Overall, the mean C accumulation rates of the peat cores decreased with depths with one exception, the maxima of the mean C accumulation rates of the peat cores from S were observed in the 60–100-cm section (Table 1).

**Table 1:** Soil properties, including the AMS dating result and carbon accumulation rate, of samples from 9 peat cores from the Sanjiang Plain.

Sample	Location	Depth	Total C (g·kg^−1^)	Total N (g·kg^−1^)	C/N ratio	pH	Bulk density (mg·cm^−3^)	AMS ^14^C age (^14^Cyr BP)	C accumulation rate (g C·m^−2^ yr^−1^)
S1	Shenjiadian1	0–30cm	340.62	21.62	15.75	5.24	0.588	863	101.37
S2	Shenjiadian2		291.15	11.94	24.38	5.38	0.535	820	84.85
S3	Shenjiadian3		316.33	11.62	27.22	5.31	0.462	637	36.98
S4	Shenjiadian1	30–60cm	349.70	30.46	11.48	5.36	0.172	1554	31.38
S5	Shenjiadian2		379.96	20.68	18.37	5.35	0.046	1903	13.92
S6	Shenjiadian3		391.92	35.58	11.02	5.37	0.417	1136	430.85
S7	Shenjiadian1	60–100cm	329.26	20.36	16.17	5.58	0.558	2239	112.10
S8	Shenjiadian2		193.44	16.87	11.47	5.61	0.549	2382	52.97
S9	Shenjiadian3		259.43	18.05	14.37	5.17	0.342	1607	39.88
S10	Shenjiadian1	100–200cm	29.74	11.88	2.50	5.81	0.704	3526	4.19
S11	Shenjiadian2		319.96	17.26	18.54	5.08	0.412	3275	15.55
S12	Shenjiadian3		269.36	14.51	18.56	5.62	0.680	3776	21.89
H1	Honghe1	0–30cm	336.08	15.01	22.39	4.98	0.390	1342	45.39
H2	Honghe2		286.88	19.11	15.01	4.91	0.577	683	70.92
H3	Honghe3		423.85	34.05	12.45	5.44	0.574	764	205.66
H4	Honghe1	30–60cm	68.95	2.95	23.37	5.32	1.181	3741	8.22
H5	Honghe2		331.76	18.19	18.23	5.56	0.428	1412	55.86
H6	Honghe3		391.40	19.04	20.56	5.65	0.407	1029	81.99
H7	Honghe1	60–100cm	283.30	9.09	31.16	5.59	0.379	6275	2.55
H8	Honghe2		254.18	7.44	34.16	5.57	0.382	2510	24.33
H9	Honghe3		141.34	5.02	28.16	5.51	0.973	3727	10.39
H10	Honghe1	100–200cm	278.36	19.00	14.65	5.60	0.382	8166	1.84
H11	Honghe2		338.80	20.74	16.34	5.60	0.380	5053	9.97
H12	Honghe3		89.23	8.59	10.39	5.73	0.384	5625	6.65
Q1	Qindelie1	0–30cm	430.12	16.10	26.72	4.80	0.335	2085	9.29
Q2	Qindelie2		382.54	15.28	25.04	5.14	0.448	924	56.74
Q3	Qindelie3		378.24	15.57	24.30	4.96	0.590	1020	61.72
Q4	Qindelie1	30–60cm	267.59	13.39	19.98	5.30	0.486	4626	14.66
Q5	Qindelie2		458.54	16.79	27.31	5.30	0.218	2088	12.99
Q6	Qindelie3		156.00	13.04	11.96	5.27	0.905	2694	0.96
Q7	Qindelie1	60–100cm	141.91	12.13	11.70	5.80	0.76	11496	0.99
Q8	Qindelie2		181.95	14.08	12.92	5.55	0.735	4915	5.44
Q9	Qindelie3		44.72	6.67	6.7	5.91	0.812	4410	4.31

(The peatlands located in Qindelie were shallower than those at the other sites, with a depth of 100 cm).

A summary of the soil physico-chemical characteristics is presented in Table 1. Soil pH was consistently acidic and varied from 4.80 to 5.91. Soil total C (TC) and N (TN) ranged from 29.74 to 458.54 g/kg and from 2.95 to 21.62 g/kg, respectively. Overall, they were decreased with depths at all sites, and the soils from S, the youngest fen, showed the highest total C and N contents. The C/N ratio was approximately 1.5 times lower in the lowest section than in the surface section of the same site.

Soil age (analysis of variance (ANOVA), F = 7.320, p = 0.001), TC (F = 4.333, p = 0.012) and pH (F = 9.297, p < 0.001) differed vertically. However, the C accumulation rate (F = 0.330, p = 0.804), TN (F = 7.320, p = 0.216), C/N ratio (F = 1.505, p = 0.234) and bulk density (F = 0.603, p = 0.618) did not. Interestingly, we found that TN (Pearson test, r = −0.360, p = 0.039), TC (r = −0.505, p= 0.003) and the C accumulation rate (r = −0.349, p= 0.046), were negatively correlated with peat age, whereas pH (r = 0.541, p = 0.001) was positively correlated with peat age.

### Bacterial species richness and equitability

Concentrations of DNA extracted from peat soils across different sites and depths were ranged from 88.2 to 240.9 ng/µL. After amplification of the V3-V4 region of the 16 rRNA, 1,789,830 sequences from 24 soil samples were sequenced using Illumina HiSeq (Tables 2), and 1,205,425 sequences remained after quality control. After resampling with 21,811 sequences, the number of operational taxonomic units (OTUs) per sample ranged from 357 to 1542. The Shannon, Simpson, Chao1, ACE and PieLou equitability indices were calculated to estimate microbial richness and equitability (Table 2). The Shannon (ANOVA, F = 3.978, p = 0.017) and PieLou equitability (F = 4.329, p = 0.012) indices changed significantly from the surface to the bottom layer of the peat cores. However, no change was observed for the OTU number (F = 2.521, p = 0.077) or Simpson (F = 1.867, p = 0.157), Chao1 (F = 1.922, p = 0.148), and ACE (F = 1.670, p = 0.195) indices.

**Table 2:** Diversity indices of the bacterial communities of Shengjiadian (S), Honghe (H) and Qindelie (Q) fens.

Sample name	Sequenced reads	OTU number	Shannon	Simpson	Chao1	ACE	PieLou equitability
S1	25,518	1007	6.487	0.951	1194.4	1233.4	0.650
S2	39,835	1542	8.102	0.99	2152.6	2219.1	0.765
S3	41,599	1119	7.542	0.986	1472.4	1515.1	0.745
H1	50,533	1000	7.643	0.988	1285.0	1289.6	0.767
H2	45,427	1351	7.866	0.982	1549.1	1537.1	0.756
H3	25,962	1215	7.603	0.979	1413.5	1430.8	0.742
Q1	40,211	1006	6.856	0.97	1327.4	1371.6	0.687
Q2	36,181	1490	7.978	0.983	1763.1	1721.2	0.757
Q3	43,837	855	6.8	0.974	1066.3	1078.9	0.698
S4	37,855	1069	7.145	0.981	1454.2	1504.3	0.710
S5	37,750	869	6.705	0.972	1187.7	1205.4	0.687
S6	26,248	933	7.056	0.976	1113.3	1162.3	0.715
H4	44,583	973	6.425	0.92	1171.2	1176.9	0.647
H5	22,157	1275	7.855	0.985	1500.8	1513.8	0.761
H6	32,546	1216	7.502	0.978	1381.5	1474.8	0.732
Q4	41,677	1090	6.755	0.965	1568.3	1571.9	0.669
Q5	32,136	967	6.968	0.976	1130.4	1217.5	0.703
Q6	44,614	357	4.498	0.828	447.8	492.1	0.530
S7	25,647	1267	7.73	0.982	1468.6	1504.7	0.750
S8	47,196	725	5.921	0.953	941.8	999.8	0.623
S9	42,905	808	5.74	0.928	1174.9	1252.5	0.594
H7	37,366	1217	7.265	0.975	1556.9	1614.0	0.709
H8	38,994	933	6.482	0.969	1250.0	1284.9	0.657
H9	21,811	1363	7.524	0.981	1860.5	1870.1	0.723
Q7	39,687	921	6.957	0.978	1245.1	1260.8	0.707
Q8	27,396	594	6.293	0.969	696.6	716.6	0.683
Q9	27,489	1208	7.809	0.98	1372.7	1399.0	0.763
S10	39,499	596	4.952	0.898	814.0	887.1	0.537
S11	31,287	833	6.942	0.979	973.0	1031.8	0.716
S12	45,560	704	5.24	0.92	919.9	988.9	0.554
H10	35,835	821	6.145	0.954	1177.3	1149.6	0.635
H11	38,421	1040	6.324	0.95	1336.2	1409.3	0.631
H12	37,663	988	6.482	0.957	1307.3	1339.9	0.652

### Microbial community composition

**Figure 2:**
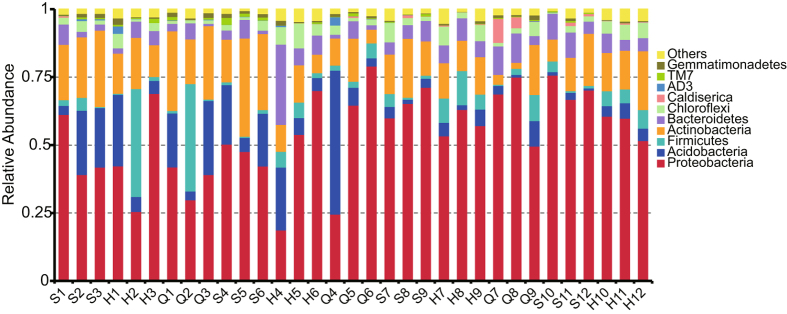
Bacterial community structure variation in the Shengjiadian (S), Honghe (H) and Qindelie (Q) fens. The relative abundance of bacteria at the phylum level is shown. Each bar represents the relative abundance of each sample. Each color represents a particular phylum. The numbers associated with the sample names indicate the sampling depth.

**Figure 3:**
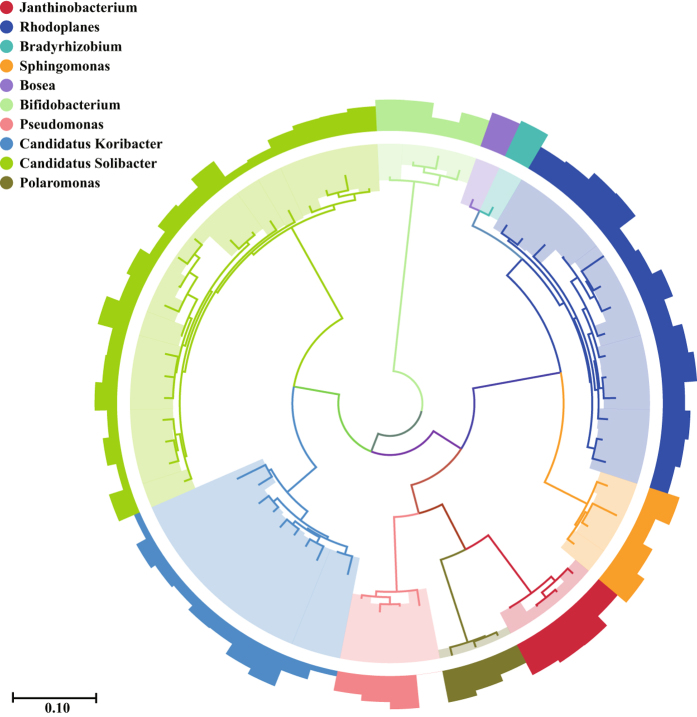
Phylogenetic tree of the operational taxonomic units (OTUs) belonging to the top 10 genera. Each color represents a particular genus. Each bar represents a particular OTU. The second levels indicate the relative abundance (logarithm) of the corresponding OTU. The highest relative abundance is 22.47%. Phylogenetic tree and bar chart were matched via Scalable Vector Graphics (1.1). Phylogenetic tree with bootstrap values can be found in Supplementary Figure 2. The relative abundance of each OTUs can be found in Supplementary Table 2.

Diverse bacteria were found at different peat depths. OTUs were affiliated with of 55 bacterial phyla, with the 10 most abundant phyla being Proteobacteria (54.29%), Actinobacteria (16.07%), Acidobacteria (10.10%), Bacteroidetes (6.06%), Firmicutes (5.57%), Chloroflexi (3.30%), Gemmatimonadetes (0.81%), Caldiserica (0.57%), TM7 (0.51%) and AD3 (0.28%) (Fig. 2). The bacteria ranking in the top 10 in terms of abundance at the genus level belong to Acidobacteria, Proteobacteria and Actinobacteria (Fig. 3).

### Community comparison across all sites and depths

**Figure 4:**
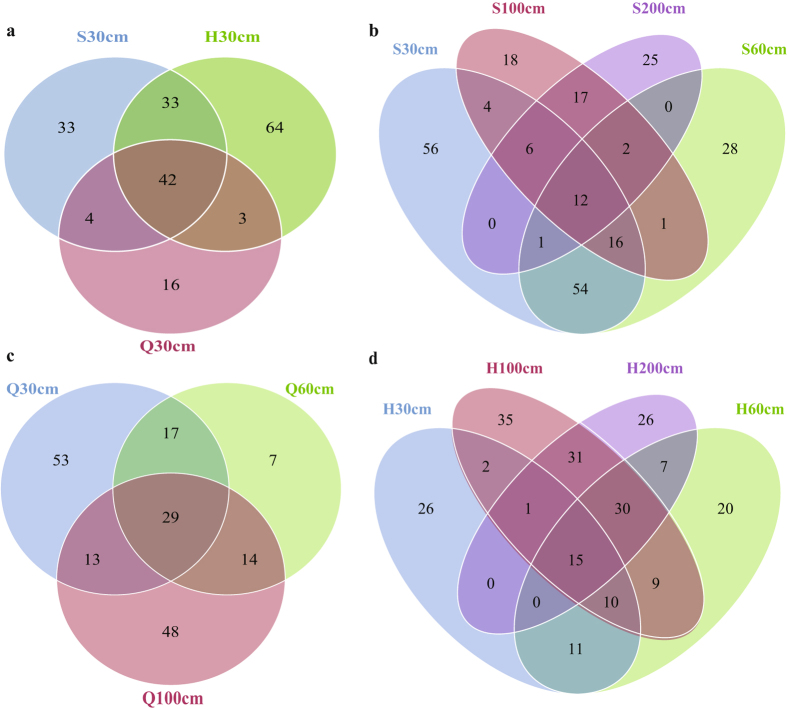
Venn diagrams showing the unique and shared OTUs of the bacterial communities across different sites (**a**) and depths in fens S (**b**), Q (**c**) and H (**d**) fens. Only OTUs with a relative abundance higher than 0.1% and detected in more than 6 samples are used for this statistical analysis.

A Venn diagram was used to compare the similarities and differences among the communities in the different samples. Because they were more likely to reflect community function, only highly abundant OTUs (relative abundance higher than 0.1% and detected in more than 6 samples) were used for the calculations. The bacterial communities of the surface peat of the S, H, and Q fens shared 42 common OTUs (Fig. 4a), and bacterial communities varied with depth at each site. For example, in the soil sampled from S, the unique OTU numbers of the 0–30, 30–60, 60–100, and 100–200-cm sections were 56, 28, 18, and 25, respectively (Fig. 4b).

**Figure 5:**
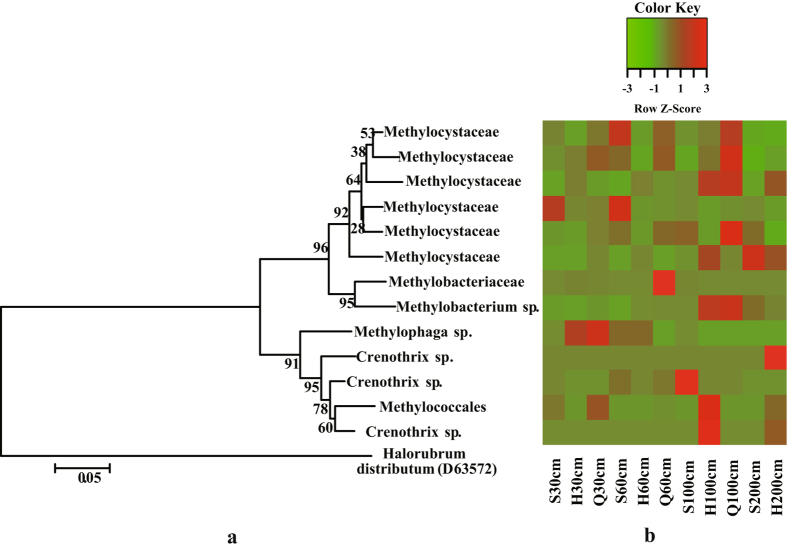
A phylogenetic tree (**a**) and distribution patterns (**b**) of methanotrophs retrieved from fens S, H and Q.

Bacterial communities differed across depths and sites. The relative abundance of the dominant phylum, Proteobacteria, increased with increasing depth across all sites and was higher in younger peatlands, in contrast, Actinobacteria and Acidobacteria sequences decreased with increasing depth (Fig. 2). From top to the bottom, the relative abundance of Actinobacteria in the peat cores from S, H and Q fens, decreased from 23.60% to 13.16%, from 15.10% to 12.62%, and from 24.24% to 8.06%, respectively, and that of Acidobacteria decreased from 16.29% to 1.53%, from 12.21% to 4.72%, and from 16.78% to 4.50%, respectively Notably, a large diversity of methanotrophs affiliated with Alpha- and Gammaproteobacteria was detected (Fig. 5). Methylocystaceae were the dominant methanotrophs across all peat samples and were primarily observed in the deep and aged soils.

### Environmental variation affects community structure

**Figure 6:**
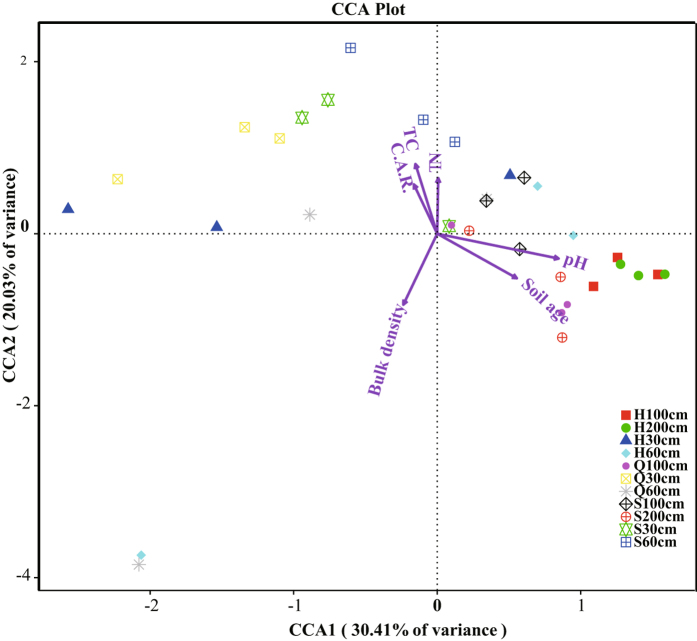
Biplot of the CCA showing the relationships between community composition and soil properties. Arrows denote biplot scores for the constraining variables, with the longest independent variable vectors being most strongly related to variation in community composition (based on OTUs clustered at 97% identity). The circles represent individual sampling sites.

We used constrained correspondence analysis (CCA) to analyze variations in bacterial community structure and its associations with environmental variables. Overall, the measured environmental variables were not significantly correlated with bacterial community structure (Mantel test, r = 0.07, p = 0.239), and only those environmental variables that showed a significant correlation (p < 0.05) with the bacterial community were plotted as vectors (Fig. 6). On the horizontal axis (CCA1, 30.41% of constrained variability), the most influential constraining variable was pH (biplot score = 0.84), followed by soil age (biplot score = 0.55), bulk density (biplot score = −0.23), TC (biplot score = −0.15), and TN (biplot score = 0.07). On the vertical axis (CCA2, 20.03% of constrained variability), the most influential constraining variables were bulk density (biplot score = −0.82) and TC (biplot score = 0.81) followed by TN (biplot score = 0.71), soil age (biplot score = −0.51), and pH (biplot score = −0.28).

### Association of bacterial diversity and composition with soil age and C accumulation rate

**Figure 7:**
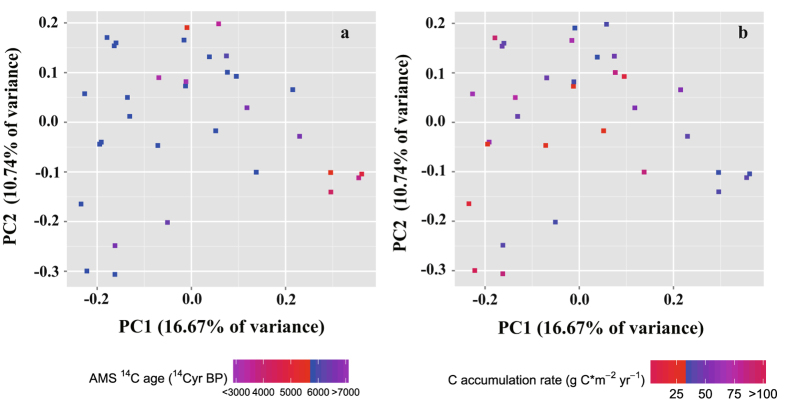
Bacterial community structures indicate by PCoA plots of unweighted UniFrac distances among sites. Sites are color-coded according to the gradients of soil age (**a**) and C accumulation rate (**b**).

Using multiple alpha diversity indices, we assessed whether soil age and the C accumulation rate are significantly related to different aspects of microbial alpha diversity. However, we observed no significant relationships, positive or negative, among these parameters (all p > 0.05). Nonetheless, principal coordinate analysis (PCoA) of unweighted UniFrac distance ordinations did show that the bacterial community structures across the 33 soil samples were affected by soil age (Mantel test, r = 0.195, p < 0.05), but not the C accumulation rate (r = −0.119, p = 0.889) (Fig. 7). In regression analysis between the PCoA scores and soil ages, a significantly linear regression with the PCoA horizontal axis was found (r = 0.701, p < 0.001), which indicating that the variation in the bacterial community could be explained by soil age along the PCoA horizontal axis. This finding is consistent with the results of CCA analysis.

## Discussion

We captured a rich microbial community from the peat cores collected from the Sanjiang Plain, at the southern edge of northern peatlands. However, we found that the bacterial community to be dominated by a few bacterial classes and OTUs that have frequently been detected in other peatland ecosystems. The most abundant phyla, Proteobacteria, Actinobacteria, Acidobacteria, Bacteroidetes and Firmicutes, have frequently been detected as dominant groups in other peatland ecosystems (oligo-mesotrophic, ombrotrophic and tundra)^5, 25, 26^.

Due to the vertical gradients in the soil properties we measured, such as TC, TN, pH, bulk density, and C/N ratio, as well as the availability of oxygen (O_2_) and other electron acceptors, such as SO_4_^2−^ and, NO_3_^−^, mentioned by others^5, 27^, the peat soil provides vertically stratified heterogeneous habitats for bacteria. In the present study, bacterial alpha diversity and composition varied as a function of peat depth and its associated physico-chemical properties, and pH, TC, TN, and bulk density showed significant effects on bacterial community structure.

The dominant microbial taxa detected at the surface are members of Acidobacteria, Gammaproteobacteria, Alphaproteobacteria (Acetobacteraceae), and Verrucomicrobia (Chthoniobacter). Acidobacteria, which are reportedly dominant among soils rich in organic matter, are involved in degradation of lignocellulose and cellulose^28–30^. A substantial number of carbohydrate-utilizing Acidobacteria and Alphaproteobacteria from acidic peatlands have been cultivated under aerobic conditions^31^. *Candidatus Solibacter*, the most abundant taxon among our samples, has been shown to be able to break down, utilize, and biosynthesize of diverse structural land-storage polysaccharides and exhibits resilience to fluctuating temperatures and nutrient-deficient conditions^32^.

According to the research of Xing *et al*.^17^, potential peatlands cover an area of approximately 10,520 km^2^ on the Sanjiang Plain and currently store ~0.26 Pg C. In response to future climate change and CO_2_ fertilization, both net primary productivity and CH_4_ fluxes will increase^33^. Methanotrophic bacteria can act as a natural barrier capable of significantly reducing the flux of methane into the atmosphere^34^, and bacteria that perform aerobic and anaerobic methanotrophy were detected in our samples. Although the relative abundance of type I and type II methanotrophs detected in the present study was similar to that in other peatlands^25, 35^, a broader sequence diversity (14 OTUs) of putative methanotrophs belonging to Alpha- and Gamma-proteobacteria was observed. The OTUs detected in this study, are most likely to act as key players in methane cycling in this environment. Further studies on the isolation and characterization of these microorganisms are needed and will facilitate our understanding of their physiological functions and ecological importance.

By comparing the AMS dating and soil properties of the cores of peat that developed at different ages, we found that soil properties, such as TN, TC, C accumulation rate and pH, were correlated with peat age. These peatlands initiated at different times, were affected by the climatic conditions, such as temperature and precipitation, etc, and underwent different initiation and decomposition processes, resulting in the respective soil properties. It is worth noting, that we also found that bacterial community turnover (beta diversity) to be significantly correlated with soil age, bacterial alpha diversity was not. We suggest that the effect of soil age on the bacterial community was due to its correlation with soil properties. The structure and activity of bacterial communities involved in C decomposition and release processes can directly affect the C accumulation in peatlands and other ecosystems^4, 5^, Nonetheless, when investigated the linkage of organic C accumulation and microbial community dynamics in a sandy loam soil, Zhang *et al*. found that C accumulation promoted the macroaggregation and reduced the effective diffusion coefficient of oxygen, causing changes in microhabitats and a shift in microbial communities^36^. We did not detect significant correlation between bacterial community turnover and the C accumulation rates. The role of bacteria in C cycling in these peatlands requires further verification via field experiments and laboratory ecophysiological studies.

In summary, we found that the peatlands on the Sanjiang Plain developed over a wide range of dates during the Holocene, with the peat cores being dated from 637 to 11,496 cal. yr BP. The relative abundance, distribution, and composition of the microbial communities in three different minerotrophic fens on the Sanjiang Plain, at the southern edge of northern peatlands, was investigated by next-generation sequencing. We captured a rich microbial community that included many rare OTUs but was dominated by a few bacterial taxa that have frequently been detected in other peatland ecosystems. Notably, a large diversity of methanotrophs affiliated with Alpha- and Gammaproteobacteria was detected. In addition, bacterial alpha diversity and composition varied as a function of peat depth and its associated physical-chemical properties, such as TC, TN, pH and bulk density. We also found that bacterial community turnover (beta diversity) to be significantly correlated with soil age, though this was not observed for bacterial alpha diversity.

## Methods

### Site description and sampling

We collected peat cores from three different minerotrophic fens, Shenjiadian (S), Honghe (H), and Qindelie (Q), on the Sanjiang Plain (129°11′–135°05′E, 43°49′–48°27′N), northeastern China (Fig. 1). The three fens began to develop during different periods of the Holocene^20^. This region is a large alluvial plain that is crossed by three major rivers, the Heilong River, Ussuri River and Songhua River, and has a total area of 10.9 × 10^6^ ha, an elevation of <200 m and a slope grade of <1:10,000. Over 70% of this region is dominated by freshwater wetlands that have developed in ancient riverbeds and waterlogged depressions^37^, and approximately 30% (or nearly 3.3 × 10^4^ ha) of this region is covered by peatlands that developed under certain topographic conditions during the Holocene or earlier^38^. The present climate of the plain is a temperate humid or subhumid continental monsoon climate. The mean annual temperature ranges from 1.4 to 4.3 °C, with an average maximum of 22 °C in July and an average minimum of −18 °C in January. The mean annual precipitation is 500–650 mm, and 80% of the rainfall occurs between May and September^39^. In addition to precipitation, the fens receive water inputs from groundwater and they are primarily covered with sedges (*Carex lasiocarpa*). In May 2012, triplicate cores were collected from each fen using a Russian peat corer, the cores were subsampled for chronological, microbiological and physico-chemical analyses. The storage of the soil samples for chronological analysis is described in more detail in the work of Zhang *et al*.^18^. For the microbiological and physico-chemical analyses, peat cores were sectioned into depth intervals of 0 to 30, 30 to 60, 60 to100 and 100 to 200 cm and; homogenized in sterile bags. After subsampling, the peat soils were stored in a sample incubator with a cooling function (2 to −10 °C) before being transferred to the laboratory. Chronological and physico-chemical characterization was conducted immediately after the samples arrived. Samples for DNA extraction were immediately frozen at −80 °C.

### Chronological and physico-chemical characterization

Subsamples with a volume of 3 cm^3^ were used for loss on ignition (LOI) with sequential combustion at 500 °C to estimate the organic matter content^40^. The bulk density of a 1-cm interval of each peat core was calculated according to the dry weight and volume of each subsample. Ash-free (organic matter) bulk density was calculated from the measurements of the bulk density and organic matter contents. All subsamples for AMS dating were dated with an accelerator mass spectrometry system at the Institute of Earth Environment, CAS. The AMS ^14^C dates were calibrated into calendar ages using the program Calib 7.02 based on the INTCAL 13 calibration dataset^41^. Carbon accumulation rates were calculated by multiplying the organic carbon content (using 52% C in peat organic matter) by the bulk density and dividing by the age interval^42^. Soil pH was measured in a 1:5 soil/water suspension^43^. The total nitrogen (N) in the soil was determined by dichromate oxidation using a Continuous Flow Analytical System (SAN++, SKALAR, Netherlands).

### DNA extraction and sequencing

Genomic DNA was extracted from 0.5 g of peat soil using a FASTDNA^TM^ SPIN Kit for soil (MPBio, Santa Ana, USA) according to the manufacturer’s instructions. The DNA concentrations were measured using a NanoDrop 2000 spectrophotometer (NanoDrop Technologies, Inc., Wilmington, USA) and diluted to 1 ng/μL. The V3-V4 region of the bacterial 16S rRNA gene was amplified using the primer pair 341F (CCTAYGGGRBGCASCAG), 806R (GGACTACNNGGGTATCTAAT) combined with Illumina adapter sequences, and barcodes^44^. The specific barcodes used in this study are listed in Supplementary Table 1. PCR reactions were performed in a 30-μL mixture containing 3 μL each primer (2 μM), 10 μL template DNA (1 ng/μL), 15 μL Phusion® High-Fidelity PCR Master Mix (BioLabs, Inc., New England, USA) and 2 μL water. The following thermal program was used for amplification: 95 for 1 min; followed by 30 cycles of 98 °C for 10 s, 50 °C for 30 s, and 72 °C for 30 s; and a final extension step at 72 °C for 5 min. Each sample was amplified in triplicate, and the PCR products were pooled and purified using Qiagen Gel Extraction Kit (Qiagen, Hilden, Germany). Amplicon-based sequencing libraries were generated using the TruSeq® DNA PCR Free Sample Preparation Kit (Illumina, San Diego, USA) according to the manufacturer's instructions and pooled at an equimolar ratio. The Illumina HiSeq2000 platform at Novogene Bioinformatics Technology Ltd was used for 250-bp paired-end sequencing.

### Sequence data preprocessing and statistical analysis

Raw sequences were divided into sample libraries via sample-specific barcodes and truncated after cutting off the barcode and the primer sequence. Forward and reverse reads with at least 10-bp overlaps and less than 5% mismatches were merged using FLASH^27^. Quality filtering of the raw tags was performed according to the QIIME (V1.7.0, http://qiime.org/index.html) quality control process^45^, and all sequences shorter than 200 bp in length and with an average quality score lower than 25 in the raw reads were removed. The remaining sequences were subjected to chimera removal using the UCHIME algorithm (http://www.drive5.com/usearch/manual/uchime_algo.html). UPARSE^46^ (version 7.0.1001, http://drive5.com/uparse/) was employed to classify the operational taxonomic units (OTUs) at the 97% similarity level. The longest sequence that had the largest number of hits to other sequences in each OTU was screened as a representative sequence. All the OTUs with sequence numbers ≤ 2 were removed in the subsequent analysis. The RDP classifier (version 2.2, http://sourceforge.net/projects/rdp-classifier/) was used to annotate taxonomic information for each representative sequence. Representative sequences were aligned using the Greengenes Database^47^ (version 2011, http://greengenes.lbl.gov/cgi-bin/nph-index.cgi), with a minimum identity of 80%. To study the phylogenetic relationships among different OTUs and the differences between the dominant species in different samples (groups), multiple sequence alignments were performed using the MUSCLE software (version 3.8.1, http://www.drive5.com/muscle/)^46^. For alpha and beta diversity analyses, the sequences were rarefied to depths of 21,811 sequences per sample, to minimize the effects of a different sampling efforts. Alpha diversity indices, including Chao1, Shannon, Simpson, ACE and PieLou equitability, were calculated. Beta diversity among the microbial communities was evaluated using both weighted and unweighted UniFrac distances. Evolutionary relationships were analyzed with neighbour-joining phylogenetic trees constructed in MEGA6. Pearson correlation analyses were applied to evaluate the relationships between the soil geochemical^48^. Differences in soil properties across samples were determined by ANOVA in IBM SPSS (version 19.0, Chicago, IL, USA)^49^. Relationships between the taxonomic diversity and the soil properties were examined by linear regression analyses using SPSS. Principal coordinate analysis (PCoA) was conducted using the ape package (version 4.1) in R (version 3.3.3)^50^. Constrained correspondence analysis (CCA) was performed using the vegan package (version 2.4–1) in R^48^. A Mantel test was used to study the relationship between bacterial similarities and overall environmental factors. We also performed post hoc permutations using the function “envfit” in the vegan package to detect associations of the microbial community composition with environmental variables. A Mantel test was also used to examine the correlation between the UniFrac distances and soil age, and C accumulation rate. Heatmap plots were generated in R using the pheatmap package^51^.

The Illumina sequencing data obtained in the present study have been deposited in the NCBI SRA database under the accession number SRP082472. The OTU table used for alpha and beta diversity analyses is provided as Supplementary Table 3.

## Electronic supplementary material

Microbial communities in peatlands along a chronosequence on the Sanjiang Plain, China

Dataset1

Dataset 2

Dataset 3
