# Supplementary material for: Microbial communities in peatlands along a chronosequence on the Sanjiang Plain, China
Source: Sci Rep. 2017 Aug 29;7:9567. doi: 10.1038/s41598-017-10436-5 (PMC5575048; doi:10.1038/s41598-017-10436-5)

Title:
Microbial communities in peatlands along a chronosequence on the Sanjiang Plain, China
Authors:
Xue Zhou, Zhenqing Zhang, Lei Tian, Xiujun Li, Chunjie Tian

Figure S1. Line chart showing the soil age of each peat core.

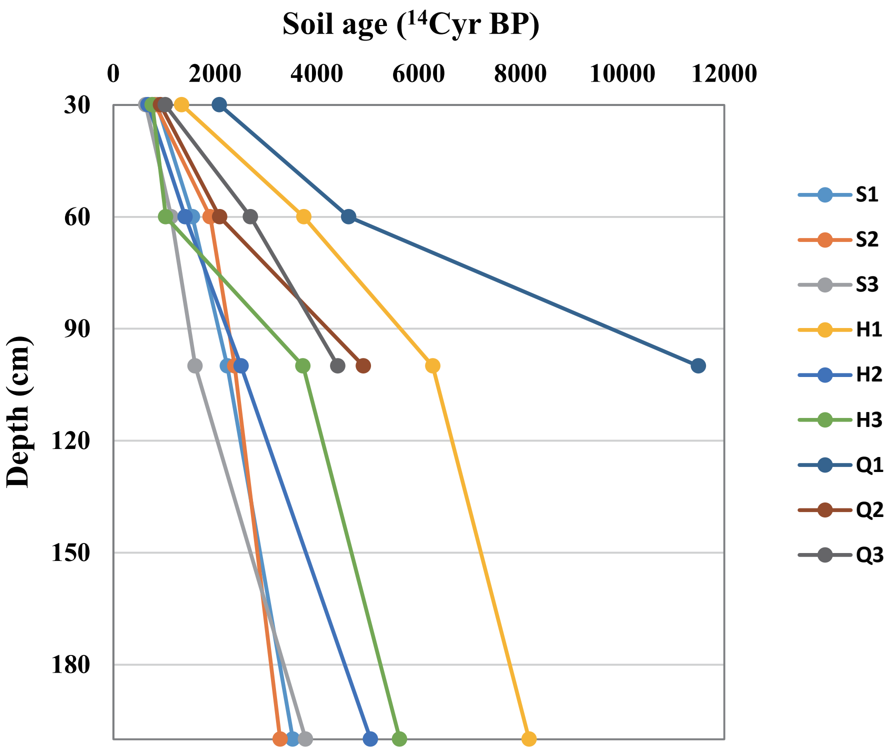

Figure S2. Phylogenetic tree of the operational taxonomic units (OTUs) belonging to the top 10 genera.

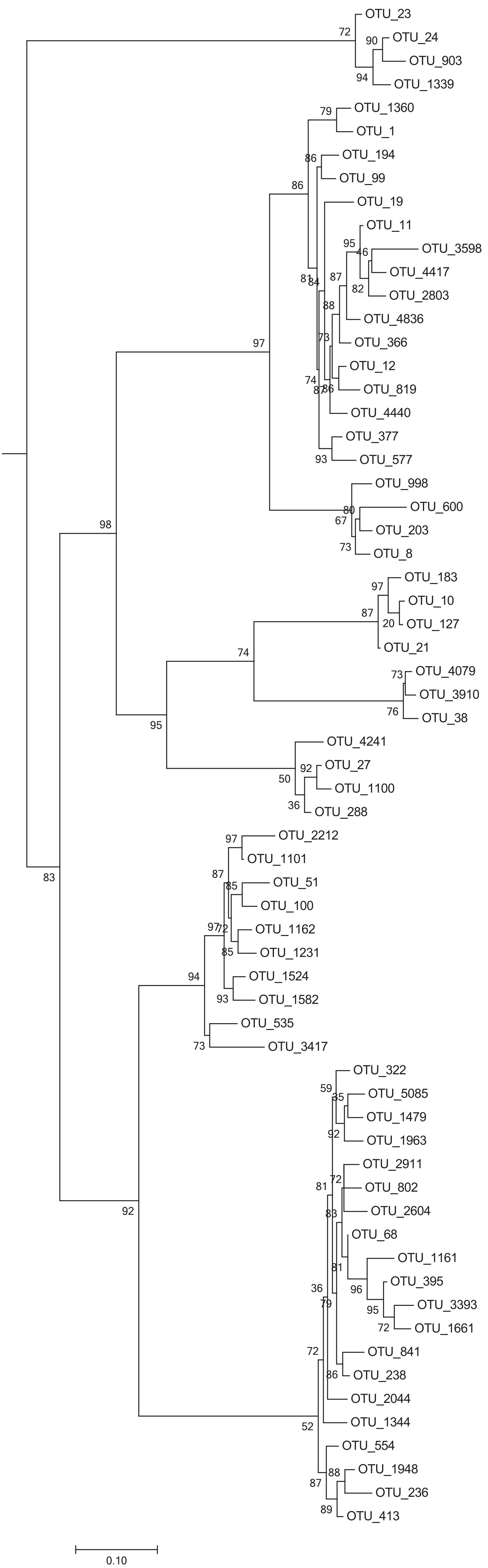

Supplement: Supplementary file 1 — Microbial communities in peatlands along a chronosequence on the Sanjiang Plain, China [file 41598_2017_10436_MOESM1_ESM.pdf]
